# The Practice of Medicine: A Treatise on Special Pathology and Therapeutics

**Published:** 1848-05

**Authors:** 


### REVIEWS.

---

ART. IV.—*The Practice of Medicine: A Treatise on Special Pathology and Therapeutics.* By ROBLEY DUNGLISON, M.D. Professor of the Institutes of Medicine, &c., in the Jefferson Medical College, Philadelphia; Lecturer on Clinical Medicine, etc. Third edition—2 vols. Philadelphia: Lea & Blanchard. 1848.

*A Treatise on the Practice of Medicine.* By GEORGE B. WOOD, M. D., Professor of Materia Medica and Pharmacy in the University of Pennsylvania; one of the authors of the Dispensatory of the United States of America, etc. 2 vols. Philadelphia: Grigg, Elliott, & Co. 1847.

In placing these two works at the head of this article, it is not our design either to draw a parallel between them or to analyse both of them. The treatise of Professor Dunglison has been before the profession for sometime. We may therefore pass it by, merely observing that the industrious author has availed himself of all the recent advances in medical pathology and therapeutics in order to make this edition a truthful reflection of the present state of medicine. It is certainly the most complete treatise of which we have any knowledge. There is scarcely a disease which the student will not find noticed.

The treatise of Professor Wood being the more recent is less known, but is not less deserving of estimation, and we shall devote a few pages to a cursory notice of it. In some respects it differs from most of the treatises that have preceded it. It is divided into two parts: part first, constituting about one-third of the first volume, is occupied with the subject of general pathology and therapeutics. In the second part —that of special pathology—the diseases belonging to the obstetrician and surgeon in common with the physician—viz: diseases peculiar to the female, and those of the eye, ear, and male generative organs—are not noticed by the author; but by way of compensation there is a much fuller discussion of other subjects. Again Dr. Wood does not hurry over what are considered the less important diseases; he observes very correctly, we think, "that every disease which is at all worthy of notice should be well understood."

In the first part the author speaks first of "the constituent forms of disease, or of those derangements which, by their various combination, constitute diseases as we ordinarily see them;" secondly of etiology; thirdly of symptomatology; and lastly of the general principles of treatment, or general therapeutics. All these chapters contain much that is valuable, but which we cannot present in a way to be of service to the reader and to do justice to the author. A few points only can be noticed by us.

All diseases may be referred to the fluids or solids of the body, or to both. The blood may be considered as the only fluid subject to original morbid changes. It is the source of all the fluids, except the lymph and chyle, and as these enter directly into its constitution they convey into it all the deleterious properties with which they are charged. The section on diseases of the blood is concise and presents about all that is known on the subject.

Diseases of the solids are considered under two divisions; one including all those that proceed from mechanical and chemical causes; the other, those arising from influences exerted on the vital properties The latter includes *irritation*, *inflammation*, *depression*, *congestion*, *fever*, and *peculiar morbid products*, as tubercle, melanosis, cancer, &c. Some of these are not elementary—fever, for example—but as it is not possible to analyze them, they may be placed among the simpler forms.

The term irritation is used by the author in its etymological sense, as signifying a state of excited or exalted action. This exaltation above the healthy standard, may be in the line of health, or more or less deflected from it, and present something peculiar or *specific*. So with depression, or, as it is commonly termed, sedation. But depression must not be confounded with debility, or diminished excitability.

"It must be borne in mind that action, the power to act, and the susceptibility to the influence of excitant agents, which is here denominated excitability, are different conditions or qualities of the system, and may each be reduced without a necessary reduction of the others. A morbid diminution of action is *depression*, a similar diminution of power is *debility*, and the term *diminished excitability* explains itself.

"These conditions may and often do co-exist; but a few remarks will show that they are distinct, and may exist separately. Thus, depression or diminished action may result from a temporary abstraction of some wonted stimulus, or the direct restrraining influence of some depressing agent, and yet the real power and excitability may be unimpaired. You may restrain a strong horse with cords, but you do not therefore necessarily lessen his strength. Loosen the cords, and it will be found that all his energy remains. Thus, remove from the system, or a part of the system, the depressing agency, and, if it has not been long applied, the unimpaired strength will be evinced by action at least as energetic as that of health. The excitability, so far from being necessarily diminished, is often increased by causes producing temporary depression; as is proved by the reaction which immediately follows the witdrawal of the depressing cause, and which is ascribable only to an augmented susceptibility, by which the ordinary healthy oxcitants produce more than their usual effect.

"That power may be diminished, without a diminution of excitability, or of action, is no less true. It is notorious, that a debilitated system is often thrown into tumultuous disorder by causes which would produce no such effect in health. Thus, in an individual much reduced by an impoverished diet, or by depletion, how often do we observe a panting respiration, and an almost convulsive action of the heart, under a degree of bodily exertion which would have scarcely discomposed these functions, in the ordinary state of the system! How often, under similar circumstances, do the slightest causes produce great nervous disturbance, amounting in the female to violent hysteria! These results can be accounted for only by admitting an increased exitability, which enables the same amount of excitant influence to produce a greater amount of effect.

"Again, diminished excitability may exist without essentially involving the existence of debility or depression. When a debilitated, but unduly excitable heart, is strengthened by suitable measures, it loses its excess of excitability as it acquires vigor; yet, by an increased application of stimulant means, it may be made to act as tumultuously as it did in its more excitable state, under a less amount of stimulation. Here then is diminished excitability, with increase of strength, and without diminution of action.

"It follows from what has been sid, that the degree of depression is by no means a certain measure of diminished strength or excitability. Yet this is often overlooked in practice, and patients with undiminished powers of system, and with perhaps an increased fund of excitability, are, in consequence of some temporary depression of the actions of health, treated with stimulant measures, to their very great detriment; and conversely, persons greatly debilitated, but with excessive action of some important organ, dependent upon increased excitability, are no less injuriously depleted with a view to the reduction of the excitement. There is no distinction more important to the young practitioner than that which I have here attempted to draw."—(Vol. i, p. 60.)

Irritation leads to inflammation, which is also considered as a state of unhealthy excitement. Dr. Wood, in one of the longest articles in this part of the work, has given a rapid but very good sketch of the progress, phenomena, effects, etc., of the inflammatory process: but he does not tell us with precision, or anything approaching to it, where he considers irritation to end and inflammation to begin.

Congestion is merely a concomitant of one of the three states mentioned, and receives a separate consideration only because of its frequent occurrence and the importance attached to it by modern pathologists. The author does not fail to insist upon the necessity of looking beyond the congestion to the cause producing it.

Fever is an important constituent of many diseases, and though not elementary may be classed among the simpler forms, as it is incapable of analysis. Our author believes in the existence of idiopathic and symptomatic fevers, but at the same time asserts that the febrile movement in the two cases is essentially the same—an assertion which some pathologists deny. The general view given in the essay is well worth the reader's attention. We make an extract from the conclusion, to show in some degree the difficulties hanging over the subject, and the fairness and candor with which the author meets them.

"Admitting fevers to be in some instances idiopathic, in others symptomatic, the proper febrile movement, from whatever cause it may proceed, and however it may be complicated in different cases, is of the same essential character. The functions are deranged, and in such a manner that all recognize the disorder when they encounter it. There must, therefore, be something, in the different febrile diseases, common to the whole of them, and to this the name of fever properly belongs.

"It is an interesting question what is the nature of the derangements in the functions. The mere statement that they are universally disordered gives no definite notion of the disease. But it is by no means an easy task to explain the nature of the derangement. Some of the functions are in a state of irritation, some probably of depression; but if we attempt to review the whole of them, and point out which are in the one state, and which in the other, we find that in many instances the syptoms do not afford us grounds for a positive decision; and we can derive no aid from organic changes; for these, when they do occur, are not essential. The order in which those states succeed each other, and the chain of mutual dependence which connects them, equally elude our search. It is possible that there may be something peculiar in the irritation and depression. But if such peculiarity exist, we cannot point out its nature. Together with disorder of the solids, there is very frequently an altered state of the blood; but the question has not yet been settled, whether this is an essential part of the disease, and important in the chain of causation, or a mere incidental effect. We are thus in a condition of uncertainty upon almost all points.

"Theorists have failed in endeavoring to trace the complicated disorders of fever to some common source, and to point out a particular succession, a particular and necessary line of march, in the progress of the affection. The universal disturbance of function which constitutes the disease may be brought about in various ways; and the starting point may be entirely different in differen cases. Yet, among the great majority of cases, there is a close analogy in the mode of onset, which must be ascribed to some common principle. Whether the fever is idiopathic or symptomatic, the first decided step towards its formation, seems to be some morbid impression upon the nervous system, and this impression seems to be of a depressing nature. The phenomena immediately preceding, and those attendant on the chill, are for the most part unequivocally those of depression. The whole nervous system appears to have received a shock from the cause, cramping, and for a time deadening its energies. Something analogous to this we have in the effects of a severe surgical operation, of a severe injury in any part of the body, and of sudden alarming or afflictive intelligence. Along with the diminished exercise of the nervous function, is necessarily a diminution of all those functions dependent upon it. We may thus partially explain the condition of the chill; but there is something more which we do not fathom; something in which the chill of fever differs from other instances of nervous depression. Upon principles which have already been explained, the general prostration is succeeded by reaction, and the fever is then established. But there is here also something more than mere reaction. There is the continued action of the cause, a diversified play of sympathies in one case, a widely pervading influence from some unknown agent in another; and fever is not purely, as some have maintained, the *resilience* of the bowed down system. A proof of this is, that the febrile excitement is by no means proportionate, in all cases, to the initial nervous depression. To unraval this complicated web is in the present state of our knowledge impossible. We are too little acquainted with the precise reciprocal action of the organs, with the nature of the nervous power by which this reciprocal action is maintained, and, in many instances, with the cause of the disease and its mode of operation, to enable us to advance far beyond conjecture. We know that the heart, arteries, and capillaries are in a state of super-excitement; that, under the combined influence of the nerves and bloodvessels, calorification is increased; that all the secreting surfaces, including that of the stomach, and all the secreting glands, and the whole process of nutrition, are deranged; sometimes probably being irritated, sometimes depressed, and sometimes peculiarly affected, under the influence of peculiar causes.

"But in many instances of fever, even the imperfect concatenation above described is not evinced. Not unfrequently, the stage of chill is so slight as to preclude any idea of its agency in inducing the succeeding stage, and sometimes it is entirely wanting. In these cases, we must suppose the various derangements constituting the fever to be induced by the exciting cause, withont a preliminary action specially upon the nervous system. Either the cause enters the circulation, and reaches all the organs so as to act on all simultaneously, or it affects some one part primarily, from which an influence is conveyed sympathetically over the system."—Vol. i, pp. 104, –6.

Article sixth treats of *peculiar morbid products*, such as tubercle, melanosis, scirrhus, encephaloid, etc., and pleases us less than any article we have yet noticed. Parasitic animals are also treated of in this section. In speaking of these, Dr. Wood does not favor the theory of spontaneous or equivocal generation, but thinks that they are derived from germs or ova of preceding animals of the same species. Such is nature's general law, and, until it can be shown to be insufficient or inapplicable, correct philosophy requires all uncertain cases to be referred to it. In the present instance no such inapplicability exists, as all the difficulties are easily explained.

"Supposing the entozoa to have the same origin as other living beings in general, their germs must be derived from one of two sources; either they must have been received from the parent, and thus pre-existed in the body from birth, waiting for circumstances favorable to their development, or they must have been derived from without. In fa or of the former notion, is the fact that each species is confined. as a general rule, to a particular animal, like a plant to certain districts of country. But the explanation of this fact simply is, that the germ finds only in the particular animal in which it flourishes the condition of things favorable to its vital activity. A strong argument against the opinion, is the difficulty of supposing that the ovum or germ should have so long remained concealed in the same individual and his predecessors. possibly for many generations, without undergoing development. The probabilities are greatly in favor of its external origin. It is said that the parasitic animals of the human frame have no prototype in nature. This is true of most of them, so far as observation has extended. But, in the first place, the fact that no such prototypes have been discovered is no proof of their non-existence; and, secondly, animals of the same species as the parasite may be constantly before us in the outer world, but with characters so different that they cannot be recognized. It is highly probable that living beings, developed from ova in the human body, would be so modified by the peculiar circumstances of their position as to lose specific characteristics, which naturalists ordinarily deem essential. The eyeless fish of the Mammoth Cave of Kentucky are said by persons quite competent to decide, to be identical with those in the ordinary streams of the country. If an animal can thus, in its development from the ovum, lose an eye where it is not wanted, it may well lose other less important characters of mere form. It may well be, therefore, that of the innumerable germs which fill the external world, some may find admittance into our frames, and there expand into beings, apparently different from anything around us. But it has been objected further, that not only are these parasites found in parts of the frame having an external communication, but also in closed cavities, in the interior of parenchymatous organs, and even in the fœtus. In reply, it may be said, that wherever a blood corpuscle can circulate, there also may pass a living ovum; for the corpuscle, or at least a cell not larger than it, has within it the powers requisite for organization. Microscopic germs may enter our systems through the lungs or stomach, and circulate everywhere with the blood, and even pass along with nutriment into the fœtus, though much larger in proportion to the resulting animals than an acorn to the oak. A positive argument in favor of the opinion here advocated, is the occurrence of certain entozoa only in certain regions of country. Thus the guinea worm is known only in certain parts of the East, and of the two species of tapeworm, one is confined chiefly to certain parts of Europe, where the other is camparatively little known."—(Vol. i, p. 122.)

The chapter on general etiology, occupying thirty-five pages, contains valuable matter, but we must hurry over it with this general commendation. The next chapter, on symptomatology or semeiology, comprises a fund of information especialy valuable to our younger readers. These will excuse the length of the quotation we are about to make in reference to the symptoms presented by the tongue. We recollect the great difficulty that we used to have in forming any clear notions of the indications presented by this organ; and we take it for granted that most physicians have in the outset experienced similar difficulty.

"In drawing inferences from the condition of this organ, it is important to know, whether the appearances it may present are the result of local disease in the mouth, or of the sympathies which connect it with other parts of the system. In general, there is little difficulty in coming to a correct conclusion upon this point; and it is only necessary that the attention should be directed towards it. The following remarks have reference only to the general indications offered by the tongue. This organ seems to have been designed as an index, to the eye as well as to the ear, of the state of the system; so numerous and diversified are the morbid affections which modify its healthy appearance. It not only participates in all general derangements of the whole system, serving as one of the surest guides to a correct judgment in relation to the degree, progress, and precise stage of the disease; but especially also sympathiszes with the different parts of the digestive tube, at one extremity of which it is placed.

"The *bulk* of the tongue may be increased or diminished. Its enlargement, when not so considerable as to be very obvious, may often be known by the appearance of indentations on its sides, made by the pressure of the teeth. This is occasionally one of the first signs of the mercurial influence. Its contraction, when not the mere effect of dryness, is usually the result of a diminished supply of blood, and indicates either a general deficiency of the circulating fluid, or great feebleness of the heart's action. Like every oth r part naturally moist, it shrinks by drying; and, under such circumstances, no general inference can be deduced from its mere loss of volume.

"Its *color* is often greatly and significantly modified. Morbid floridness of the tongue is the consequence either of the condition of the blood, or of its greater abundance in the organ In the former case, an unduly arterialized state of the mass of the blood is indicated; in the latter, either over-excitement of the circulation or generally, phlogosis of the stomach. Redness of the tongue, not the result of local causes exclusively, has been supposed by some pathologists to be an almost certain sign of gastric inflammation or irritation. But this is far from being the case. It is often seen when no evidence of gastritis is presented, either by the symptoms or upon dissection, and is not unfrequently absent when that disease exists. Serious practical injury may result from this error. The red tongue can be considered as having special reference to the stomach, only when other symptoms point in the same direction, and even then, is by no means a certain sign. A *livid* or *purple* color of the tongue is usually dependent upon an insufficient aëration of the blood, and is a valuable sign, in connexion with the same color of the lips. Not unfrequently the tongue is morbidly *pale;* and this state is a sign of deficiency of the blood in general, or of its red corpuscles in particular, or of great prostration of the circulating forces.

"Its condition as to *dryness* and *moisture*, is often highly significant. But caution is necessary not to mistake dryness from temporary and unimportant causes, for that which results from general disease. In persons who sleep habitually with their mouths open, the tongue is apt to be dry in the morning; and the same cause often produces the same effect in sickness. On visiting a patient, we find the tongue unexpectedly dry, and begin to feel some apprehension, until we learn that the patient has been breathing for sometime through the mouth alone. A stoppage of the nostrils often gives rise to this phenomenon. In all doubtful cases, it is only necessary to request the patient to close his mouth, and then move the tonge about so as to moisten it. If he succeed satifactorily, we may conclude that the dryness was accidental, and of no account. Another caution is requisite; to take care, namely, that a really dry tongue should not be mistaken for a moist one, in consequence of the patient having recently taken a liquid into his mouth. Dryness may exist in different degrees, from mere clamminess to perfect aridity. It depends on a deficiency of saliva, or of mucus, or both, and indicates a general tendency to diminished secretion. It not unfrequently occurs, as a sympathetic affection, in ulcerative inflammation of the small intestine. It affords sometimes the most important therapeutical indications, as will be fully shown hereafter.

"The *temperature* of the tongue serves as a guide to that of the body generaly. When cold, it evinces for the most part, great prostration of the powers of life. It proves that the process of calorification is failing at the very fountain; for the breeth must be cool, before the tongue can become so in any considerable degree. This coldness of the tongue has been frequently noticed in severe cases of epidemic cholera. But we must take care not to confound coldness from local causes, as from ice in the mouth, or from the patient having slept long with the mouth open in a cold atmosphere. with that proceeding from the state of the system. Heat of the tongue, except when arising from inflammation of the organ, may be considered as a sign of a general elevation of temperature.

"But the condition usually denominated a *furred tongue*, is perhaps the most valuable diagnostic symptom afforded by that very important little member. In this state, the tongue is covered with a morbid coating, which adheres so firmly that it cannot be removed, without removing a portion of the surface along with it. Occassionally, deposits take place from the saliva; but these are easily removable, and must be distinguished from the genuine fur. The latter proceeds from a secretory process of the tongue itself, and seems to be incorporated with the superficial layer of its mucus coat. It is almost always confined to the upper surface, where the structure of the membrane is papillary. Though very generally a sign of disease, it is not always so. Some persons have a furred tongue habitually, more especially upon rising in the morning; and, though in the greater number of these, there is probably some chronic disorder of digestion, yet in others the health appears to be perfect.

"A furred tongue almost always accompanies fever, and is one of the most decided characteristics of that affection. Indeed, when considerable in degree, and not dependent upon stomatitis of any kind, it may very generally be regarded as a febrile symptom. When the fur is *white*, *thickish*, *tolerably uniform*, and accompanied with moisture, it usually indicates an open, active state of fever, in which, though the obvious symptoms may possibly be violent, there is not apt to be any lurking mischief, nor any malignant tendency. When *short*, very *adhesive*, and *rather scanty*, permitting the redness of the tongue to appear through it, and attended with some disposition to dryness, it is often a sign of a protracted and obstinate form of fever, which is apt to assume a low, nervous, or typhoid form. *A yellowish hue* of the fur is usually indicative of bilious disorder, being produced either by the vomiting of bile, or, what is probably much more frequent, by direct secretion from the tongue, consequent upon deficient secretion by the liver, or an excessive production of bilious matter in the blood. Not unfrequently, this color of the tongue is accompanied with a bitter taste. It is common in miasmatic fevers, and hepatic diseases. *A brown or black tongue*, is usually indicative of a low state of the system, and an impaired condition of the blood. It is owing to the secretion of a dark matter, apparently identical with that which collects about the teeth and lips in typhus fevers; and probably consisting of blood modified in its passage out of the vessels. The same action would seem to take place in the tongue, as that which, in the stomach and bowels, occasions the black discharges so common in malignant fevers. It may depend on an enfeebled state of the secreting tissue, or a diseased state of the blood, or on both united. Very frequently, this darkness of the tongue supervenes upon a previously white coating, and indicates a deteriorated state of the vital forces, and probably of the blood. The caution should be observed not to confound this discoloration with that which may proceed from accidental causes, as from the chewing of liquorice, tobacco, burnt coffee grains, &c. In many instances, the white fur of the tongue is modified by *red points*, which are the tops of the swollen and projecting papillæ. This appearance is not uncommon in eruptive febrile diseases, especially scarlet fever and measles. When consequent upon a dyspeptic state of the stomach, the fur is most copious in the morning before breakfast. In some persons, emptiness of the stomach is said always to induce this state of the tongue.

"The manner in which a furred tongue becomes clean affords valuable indications. When the fur slowly recedes from the tip and edges, thinning gradually as it retires, it intimates a favorable convalescence. A portion of fur often lingers near the root of the tongue, long after the disease has given way. In another mode of cleaning, the fur loosens and separates in flakes, often beginning at the middle or near the root, sometimes in large patches, or over almost the whole tongue at once, leaving a smooth, red, glossy surface, as though the papillary structure had been lost. In such cases, if acute, and if the tongue remains moist, convalescence almost always takes place, though usually tedious, and sometimes very lingering. In threatening fevers, it is very desirable to witness this phenomenon; and, as it is often preceded by a feeling of soreness in the fauces, this may be considered, when it occurs in such cases, as an auspicious circumstance. Much stress was laid upon this as a prognostic symptom by the late Dr. Joseph Parrish, of Philadelphia. Sometimes the fur recurs once, and again, before it ultimately disappears; and weeks and even months are occasionally consumed, in the struggling and apparently uncertain advance of the system towards health. In less favorable cases, the tongue, after having commenced the process of cleaning, as just described, or even after completing it, instead of continuing moist, becomes as dry as a chip, with an aggravation of all the symptoms, and no little increase of danger. The indication is still more unfavorable, when, in addition to its dryness, the surface becomes gashed, chapped, or fissured, or exhibits a rough, scaly appearance.

"This smooth, red, and glossy state of the tongue. sometimes with moisture, and sometimes with dryness, is not uncommon in chronic diseases, in which it is generally a bad sign, and is supposed to indicate serious organic derangement of the alimentary mucous membrane. A still worse condition, however, is an aphthous state of the tongue, which is apt to come on in the advanced stages of chronic diseases, and is generally to be received, under these circumstances, as a fatal sign, though of itself, and occurring in ordinary health, it is in no degree alarming.

"A *loss* or *depravation* of *taste* is not uncommon, and is generally of little consequence, depending upon a mere derangement of the surface which receives the gustatory impressions. But, when of a paralytic nature. it is much more serious, as it generally indicates disease within the encephalon.

"The only other point requiring consideration refers to the *movements* of the tongue. When, in acute febrile diseases, these are not under the control of the patient; when, upon being requested to protrude the tongue, he is unable to do so; or when the organ trembles much in the attempt, the symptom is exceedingly unfavorable, indicating either very great general prostration, or dangerous cerebral disease. Of similar unfavorable prognosticati n, under the same circumstances, is the occurrence of a difficult and hesitating utterance, like stammering. The inclination of the tongue towards one side, when protruded, usually indicates palsy, and is one of the common attendants upon hemiplegia."—(Vol. i, pp. 162–6).

Passing over the chapter on general therapeutics. we come to the second part of the treatise, viz: special pathology and therapeutics. Here the author arranges diseases into three great classes: the first includes general diseases, which affect all the functions at the same time; the second, constitutional diseases, which may display themselves locally, but not every where at the same time; and the third, diseases affecting some particular organ or structure, and where the general affection, if any is present, is strictly secondary.

In the first class are placed the idiopathic or essential fevers—diseases having in common a febrile movement. Symptomatic fevers belong to the third class, the fever being secondary to the local affection. The idiopathic fevers are divided by the author according to the cause—thus:

1. Irritative fever; 2, miasmatic, or bilious fever; 3, yellow fever; 4, enteric or typhoid fever; 5, typhus fever; 6, plague; 7, small-pox, or variola; 8, vaccine disease, or vaccina; 9, chicken-pox, or varicella; 10, measles, or rubeola; 11, scarlet fever, or scarlatina; 12, erysipelas.

Under the term irritative fever, are included all cases of idiopathic fever resulting from causes of irritation which have nothing peculiar or specific in their mode of action, but which produce an over-excitement in one or more functions of the body. This over-excitement being propagated to different parts of the system, may throw all the functions into a state of derangement capable of sustaining itself after the cause is removed. There must be, of course, a pre-existing disposition in the system to the febrile movement, in order that it should be thus independently sustained. The degree of febrile reaction varies greatly—ranging from the mildest to the most violent. Generally the symptoms are "a hot dry skin, frequent pulse, quickened breathing, furred tongue, headache, loss of appetite, thirst, constipation of the bowels, scanty urine and diminution of the secretions generally." The pulse is sometimes remarkably frequent, but at the same time is apt to be reduced in strength and force. Sometimes there is slight inflammation of the fauces, or of the alimentary or pulmonary mucous membrane, but not to an extent sufficient to account for the fever.

The fever may be ephemeral, or may continue two, three, or four days, or even longer. The duration cannot be previously calculated, but under proper treatment is seldom greater than five days. When prolonged beyond this the fever is apt to assume the remittent form, and to be found associated with inflammation of some part. The disease frequently occurs in children, and owing to their greater impressibility is more persistent in them than in adults. The infantile remittent of authors, when not confounded with inflammation of the alimentary canal, the mesenteric glands, the liver, or with tubercular disease of the lungs and bowels, is an example of irritative fever. It is identical with worm fever. Childhood gives nothing peculiar to the nature of the disease. It may be ephemeral at this period of life as at any other; but when persistent it exhibits more or less of the following character.

"Children from two to eight years old are most subject to the complaint. Sometimes the fever approaches gradually, sometimes occurs quite abrubtly in the midst of apparently excellent health. Indeed, the child is not unfrequently attacked after some unusual indulgence of his appetite. It is seldom that any other symptom of a chill is observed than some remaining coldness of the extremities, after the surface generally has become hot. This is perhaps owing to the circumstance, that the disease is not usually noticed until the fever has become established. When first visited, the child has a hot skin, a frequent pulse, sometimes amounting to 140 or even 160 in a minute, a furred and dryish tongue, quickened breathing, thirst, aversion for food, sometimes nausea and vomiting, and generally constipation of the bowels. There is also obvious disorder of the nervous system. If able to express himself, the child often complains of pain in the head; but otherwise, is restless and fretful, or, if quiet, is heavy and disposed to sleep. Starting in sleep, and twitchings of the tendons are very frequent, and sometimes the onset of the fever is accompanied with convulsions. After a few hours, the violence of the symptoms abates; and, on the following morning, the child is found with a cooler skin, a less frequent pulse, a moister mouth, less nervous disturbance, and a brighter and more lively expression. As the day advances, the fever again rises with similar symptoms as at first, and thus the disease continues, with daily remissions and and exacerbations, till its close. In some cases, however, the remission is much less decided than in others; and occasionally it might be difficult to determine at what period in the twenty-four hours the febrile symptoms were most violent. Throughout the complaint, the tendency to constipation generally continues; and the passages, when procured, are apt to have an unhealthy appearance and odour, being clay-colored, black, or greenish, with undigested food often intermingled, and very offensive.

Sometimes there are large fecal accumulations in the bowels. The urine is scanty, and sometimes almost or quite suppressed. The breath is often sour, and of a peculiar heavy odour, characteristic of febrile disease in children. Delirium is not unfrequent; but still more frequently there is drowsiness, amounting sometimes even to stupor or coma, which is usually most striking during the exacerbations. Occasionally, the child picks its nostrils and lips, so as to make them bleed; and in the advanced stages picks also at his clothing, or the bedclothes, or at whatever other object is near him, even at imaginary objects in the air; but this phenomenon is not confined to this form of fever, nor to childen, though a much less dangerous sign in them than in adults.

"The disease runs on variously from five to ten days, or even two weeks; but, in the protracted cases, there is always reason to apprehend the existence of positive inflammation, generally within the abdomen, to which the complaint owes its continuance and danger. Unless in consequence of the supervention of inflammation, which places the disease among the phlegmasiæ, it almost always ends in recovery. The paroxysms generally become shorter and milder; purgative medicines operate more readily, and bring away more healthy discharges; the urine becomes more copious; the heat of skin and frequency of pulse abate; the tongue begins to clean, and the appetite to return; and health is gradually re-established.

"It is not always easy to distinguish the true infantile remittent from the phlegmasiæ of the bowels. The occurrence of diarrhœa or of dysenteric stools, with a tumid abdomen and persistent tenderness upon pressure in certain parts, and less decided remissions in the fever, are the most prominent diagnostic symptoms of the latter affections. Some authors have attempted to distinguish between this and the worm fever. I believe that they are identical; worms being capable of exciting the fever in those predisposed to it, as well as any other irritant. The diagnosis between the infantile remittent and enteric or typhoid fever, will be better understood when the latter complaint has been studied. It will be sufficient to state, in this place, that, in the enteric fever, so far from a disposition to constipation, there is usually either diarrhœa, or a great facility of being acted on by cathartics, so that the least doses will often operate. There is, besides, more meteorism of the abdomen, a more regular march of the fever, and altogether a severer grade of disease than in the infantile remittent. The drowsiness or stupor which often attends the latter complaint, may sometimes lead to the suspicion of cerebral disease; but the absence of violent screams and uncontrollable vomiting, in the earlier stages, and the ordinary circumstances that the stupor increases and diminishes with the fever, that the patient can be roused without much difficulty, and that there is no great derangement of the pupil, no strabismus, nor paralysis, will generally prove sufficiently diagnostic."—(Vol. i, p 225.)

The causes are of course numerous; but the most frequent one is exposure to cold. Over-exertion in hot weather or exposure to intense heat, teething, worms, errors in diet, may be mentioned among the frequent causes.

In the adult, an efficient cathartic, refrigerant diaphoretics, and attention to regimen, generally suffice in the way of treatment. A Dover's powder at night is valuable when much general soreness exists; and a purgative containing calomel will be required, when there are evidences of hepatic derangement.

In the *infantile* cases, the author thinks that the most effectual remedy, in the early stage, is a full purgative dose of calomel.

"This cathartic appears to exercise a peculiar influence over the disease. It is certainly more efficient than other medicines belonging to the same class, which operate on the bowels no less powerfully. I have often seen the disease yield immediately to a full dose of calomel, after having been ineffectually treated with magnesia, castor oil, salts, &c. For nearly thirty years, I have habitually used this medicine in the irritative fever of children, have never seen it do harm, and have very frequently found it to put an immediate end to the disease. I believe that, if given early, it will generally produce this effect, and thus prevent the protraction of the fever in the remittent form. How it operates, is a matter of conjecture. Perhaps it may do good by its powerful influence upon the liver, promoting a free secretion of bile, and unloading the portal circulation. Perhaps it may have a peculiar alterative effect. But the fact of its utility rests not upon theory, but experience. In order to avoid the sore mouth which, in some fever cases of children, it is liable to produce when long retained in the bowels, care should always be taken to administer some other cathartic, if it fail to operate freely in six or eight hours. From two to six grains of calomel, according to the age of the patient, three or four grains being the proper dose for a child during the third and fourth year, may be given in syrup or molasses, and followed, if necessary, in the time above mentioned, by a dose of castor oil or sulphate of magnesia. Should the bowels not be well evacuated in this way, the operation of the cathartic should be promoted by enemata. In cases of obstinate constipation, moderate doses of infusion of senna, with manna, fennel seed, and sulphate of magnesia, repeated at intervals of two hours, will be found very effectual. After the bowels have been freely opened, should the fever continue, it may be treated with the neutral mixture, repeated every hour or two, in the dose of one or two fluidrachms, or, if the stomach be irritable, by a dose of the effervescing draught at the same interval. Along with the neutral mixture, when there is no sickness of stomach, small doses of antimonial wine, or of tartar emetic in aqueous solution, may be used, the quantity being so regulated as not to induce vomiting or severe nausea. The spirit of nitric ether is also an admirable adjuvant, in those cases in which there is much starting or twitching of the tendons. The warm bath is useful under similar circumstances. This treatment is especially applicable in the febrile paroxysm, but it may also be employed, with smaller doses, or longer intervals between them, during the remission.

"Should convulsions have occurred or be threatened, in addition to the sweet spirit of nitre and the warm bath, as above recommended, poultices made with bruised garlic and flaxseed meal or bread and milk, should be applied around the feet, and the back should be bathed, along the spine, with brandy heated with garlic. It may sometimes be proper to take blood from the arm, or by leeches from the temples, and to make cold applications to the head; but these are by no means necessary in all cases. The practitioner must be influenced by the degree of arterial action, and the evidences of sanguineous determination to the head. Convulsions in children are often the result of mere nervous disturbance, and do not require the lancet. When, however, they continue beyond a few minutes, or show a disposition to return, especially if there be no reason to suspect spasm in the intestines, it will be safest to take blood.

"Throughout the disease, the bowels should be kept daily opened. Magnesia, alone, or combined with one of the saline cathartics, as sulphate of magnesia, tartrate of potassa and soda, or phosphate of soda, will often be useful, not only as a laxative, but also as an antacid. Should the patient be feeble, it may be given with rhubarb. Sometimes it may be proper to repeat the calomel, though in smaller doses than at first. It would be especially indicated by whiteness or blackness of the stools. Under the same circumstances, if the alimentary canal should be delicate, blue mass may be snbstituted for the calomel.

"When the breath is sour, small doses of bicarbonate of soda or bicarbonate of potassa should be given, from four to six times a day, dissolved in water or carbonic acid water. The latter salt should be preferred, when the urine is scanty, in consequence of the diuretic properties of the saline compounds of potassa.

"Should the abdomen be painful or tender, it should be covered with an emollient cataplasm; and sometimes leeches and blistering may be necessary. In cases accompanied with vomiting, teaspoonful doses of lime-water and milk, mixed in equal quantities, and given every hour, are often very serviceable. In the same cases, aromatic poultices, or weak sinapisms should be applied over the stomach; and it may become necessary to employ an anodyne enema."—(Vol. i, pp. 227–8.)

Under the term *miasmatic fever*, Dr. Wood includes intermittent, remittent, and congestive, or, as he prefers to call it, pernicious fever. These are different forms or types of disease, produced by a common cause—miasm. We find nothing with which the reader is unacquainted in the section on intermittent fever. Dr. Wood prefers small doses of the quinine, repeated every hour or two hours, (omitting the time for sleep,) as the medicine is thus more easily absorbed, and less liable to irritate the stomach. But in the quotidian when the apyrexia is short, this method cannot be followed—the doses must be larger. We presume that few of our readers employ bleeding in the cold stage. Nevertheless they may like to hear what the author says of it.

"It has always appeared to me to be opposed to a sound pathology. The congestion of the cold stage is the result of a depressed condition of the nervous system, in which the heart and pulmonary capillaries participate. The blood, imperfectly transmitted through the vessels of the lungs, and imperfectly forwarded by the heart, accumulates necessarily in the venous system behind these points of obstruction. It is not the accumulation of blood that produces the prostration, but this that causes the accumulation. How then can the abstraction of blood lessen the difficulty? If the heart and lungs acted feebly because they were overloaded, then the removal of a part of the burthen might be useful; but the fact is, I believe, that they are overloaded because they act feebly. How then can the asserted effects of bleeding in the cold stage be accounted for? Partly, I believe, by the influence upon the imagination of the patient. I have no doubt that the mere tying of his arm, as in the application of the tourniquet, would have in a considerable degree the same effect. The loss of an ounce or two of blood is said sometimes to have produced a complete solution of the paroxysm. Is it possible to conceive, that the heart and lungs, and all the interior viscera, should have been overwhelmed by having in them one or two ounces of blood beyond their proper capacity; and is it not probable, that the solution would have equally taken place, if, with the same amount of preparation, and an equal belief on the part of the patient that everything was going on favorably, no blood whatever had flowed? Besides, a chill very often changes spontaneously, within the time that is occupied in the preliminaries and the operation of venesection. May it not be, that in many of the reported cases, the same result might have happened without the bleeding? I do not, however, assert, that the modified state of the impression of the blood upon the nervous tissue, consequent upon the loss of a portion of the impressing agent, may not sometimes hasten reaction when there is a strong disposition towards it; just as the impression of sudden cold upon the surface, already chilled, will sometimes have the same effect. But, even allowing this to be the case, there are other means of obtaining the result when desirable, which are not liable to the same objections. The loss of blood, when not necessary, should always be avoided, on account of its debilitating effects. Unless under peculiar circumstances, which have already been noticed, it is never indispensable in intermittents. There are cases in which it might reduce a prostration, but just within the control of remedies, below the point of reaction. Why then resort to it?—(Vol. i, pp. 243–4.)

The second affection in this class is *remittent fever*, or the *autumnal*, *bilious*, *bilious remittent*, &c., of authors.

Dr. Wood prefers the term *miasmatic remittent;* he classes with it *continued fever*, in so far as the latter is distinguished from typhoid fever. As sometimes the type is so doubtful that there is difficulty in distinguishing the miasmatic remittent from the intermittent; so sometimes the fever pursues a uniform course, with slight or scarcely perceptible relaxation, from the beginning to the end. Concerning the grade of fever, the author remarks:

There are two distinct modes in which we may estimate the grade of the fever; first, in reference to its greater or less violence, and, secondly, in reference to the greater or less energy of system which attends it. In both respects, bilious fever varies greatly. As regards the violence of the disease, it may be of all conceivable grades, from a mildness which scarcely confines the patient, or requires the interposition of remedies, to a severity which demands the promptest treatment to preserve life, and against which all the resources of nature and art occasionally fail. The degree too of the energy of system may be very different, and may give rise to marked varieties of the disease. The state of system may be sthenic, with the blood rich and abundant, and all the vital functions full of vigor; or it may be feble, asthenic, or adynamic, with the vital forces exhausted by previous disease, intemperance or other debilitating cause. In these two conditions, the resulting febrile action partakes of the character of the vital functions; being in the former generally open, well developed, energetic, or, to use an epithet very commonly employed by writers, inflammatory; in the latter, often feeble with deficient reaction, and a tendency to prostration, and, when the blood is at the same time depraved, with the addition of symptoms usually denominated typhous. The term inflammatory, as above used, is not entirely correct; for it is not intended to imply the existence of any positive inflammation. It only means, that there is real strength or energy as well as excitement in the vital functions. Nay, inflammation is not at all incompatible with the contrary state; for we often observe it in cases of the asthenic, adynamic, or typhus form of the disease. It is necessary constantly to bear this distinction in mind, in the treatment of bilious fever; remedies being well borne and even required in the one case, which might prove in the highest degree dangerous in the other. In very many cases, it happens that the strength of system is in an intermediate state, being neither materially elevated above, nor prostrated below, the medium standard of health. The symptoms detailed immediately below are those of cases in which the vital powers are not materially depressed.—(Vol. i, p. 256.)

The whole chapter on this fever is a very excellent one, and evidently written with care. The description of the ordinary forms of the disease, and of some of the many varieties, appear to us to be admirably drawn. We pass over all this, however, to dwell shortly on the treatment.

If the attack has come on soon after a full meal, or if there is much gastric oppression, with nausea, and ineffectual efforts to vomit, with now and then eructations of sour or sharp fluids, an emetic of the milder sort is necessary. An active cathartic is always indicated, and both theory and experience point to calomel. The bowels should afterwards be kept open by the milder cathartics. Another remedy of great importance is bleeding, but it must not be resorted to indiscriminately. It will not eradicate the fever; and the great benefit to be derived from it is in preventing or removing inflammation and local determination, but even here it must be practiced always with reference to the general energy to support the system after the blood has been lost. The indications for blood-letting are not always presented in the very onset of the disease. Inflammation or the local determinations, frequently, perhaps generally, do not appear until two or three days have elapsed. One full bleeding is generally sufficient.

"There are two conditions, sometimes occurring in remittent fever, which demand the loss of blood, though this may at first sight appear to be contraindicated. One of these is the complication of acute gastritis with excessive nausea, which, by repressing the force of the pulse, and producing coolness and relaxation of the surface, suggests the idea of great prostration, when in fact the vital energies are unimpaired, and the apparent depression is the effect of excessive irritation of one of the vital organs. It is highly important, under such circumstances, to be able to detect the real state of the disease through the mask of debility. If such a condition occur early in the case, if, upon the occasional subsidence of the nausea, the pulse resumes its force, and the skin its febrile temperature, if burning pain or great tenderness in the epigastrium clearly reveal inflammation of the stomach, and if, finally, the color of the surface do not indicate the existence of depraved blood. and malignant or typhoid debility, it may be taken for granted that bleeding is called for, and there should be no hesitation in resorting to the lancet. Not unfrequently the nausea will be found to diminish, and the pulse to rise as the blood flows. The latter event may be considered as a clear proof that the remedy was indicated. In doubtful cases, it may be proper to bleed cautiously, stopping the orifice after taking a few ounces to ascertain the effect, and, if the pulse be found to flag, and the apparent prostration to increase, then to abandon the measure altogether; but, under opposite circumstances, to resume it, and continue till the requisite quantity has been lost.

"The second condition is associated with inflammation of the brain. Sometimes, in connexion with cerebritis, the general organic actions of the system flag, in consequence of the interference of the disease with the functions of the great nervous centre, which exercises in health a controlling and supporting influence over most of the vital operations. Besides the stupor or coma, which marks the cerebral disease, there is a small, feeble, and irregular pulse, with an uncertain temperature of the surface, which might lead to the inference of debility, were the practitioner not upon his guard. As in the last mentioned case, the stage of the disease, and the general signs which indicate the state of the blood, may lead to the suspicion, that the apparent debility is nothing more than a depression produced by the local inflammatory affection. Here bleeding should be tried with the same caution as in the preceding case, being abandoned if the pulse should flag further, and persevered in, if found to increase the vigor of the circulation.

"In either of the cases above mentioned, it may be proper by sinapisms to the epigastrium or extremities, hot pediluvia, or rubefacient frictions, to call off excitement from the diseased organ. and rouse the circulation, both cardiac and capillary, before resorting to the lancet, or in conjunction with it. Not only may the nature of the case be thus more clearly developed, but the vital actions may be supported under the loss of blood, until time shall be allowed for the oppressed organ to resume its function, after the removal of its load. For the same reason, in those cases of depression dependent on or connected with nausea, opium may be employed to relieve this condition, preparatory to the use of the lancet."—(Vol. i, p. 273.)

After purgatives and bleeding, Dr. Wood places diaphoretics. In the early stages he recommends the *refrigerant* diaphoretics. When the stomach is not irritable, antimonials, especially the emetic tartar, are advantageous. He does not like the combination of nitre with the antimonials. The effervescing draught, prepared according to the U. S. Dispensatory, is specially recommended by him.

"I feel very confident, that no practitioner who has ever been in the habit of using the effervescing draught, *properly prepared*, in the treatment of bilious fever, will be disposed to abandon it. To the patient it is almost always, when given quite *cold*, agreeable and refreshing, and many call frequently for its repetition. It is one of the most efficacious means of correcting nausea and vomiting, and acts more certainly and powerfully as a diaphoretic than almost any other saline substance with which I am acquainted; tartar emetic itself scarcely constituting an exception. It sometimes occasions griping pains in the stomach and bowels, with frequent small evacuations; but this tendency may be corrected by the addition of four or five drops of *laudanum*, or about twenty drops of the officinal *solution of sulphate of morphia*, to every other dose. Indeed, this addition may often be advantageously made, independently of the effect of the draught alluded to. It tends very happily to compose nervous disturbance, and aids the draught in correcting nausea. Sometimes, it even favors the diaphoretic action.

"When there is high sthenic action and no nausea, a little tartar emetic may be added to the preparation; and in cases of nervous disorder, such as startings, restlessness, wakefulness, and general vague uneasiness, the spirit of nitric ether is an excellent adjuvant, in the quantity of from thirty to sixty drops in each dose."—(Vol. i, p. 274.)

In the advanced stages, or in asthenic cases, and especially in those associated with rheumatism, great advantage will be derived from Dover's powder, in doses of ten grains, repeated every six or eight hours. During the febrile exacerbations, when the patient is not much enfeebled, sponging or ablution with cold water is valuable.

This treatment will suffice for the milder cases, but in the more obstinate, especially if inflammation is present, and bleeding has been pushed as far as is safe, mercury must be resorted to. Dr. Wood has no fear of it when properly administered; on the contrary he advocates it strongly.

It is not necessary to give mercury in all cases of bilious fever. The great majority will do well without it. But, when the disease is violent from the outset, and does not soon show a disposition to yield to the remedies employed, or when it assumes a dangerous aspect in its course, there will always be a propriety in administering it in reference to its constitutional effects. The existence of considerable inflammation, whether in the stomach, brain, lungs, liver or spleen, which cannot be speedily conquered by direct depletion, always affords an indication for its use. In the low or typhus forms of the complaint, when the lancet is inadmissible, it is a most valuable resource. The immense quantities in which it has been sometimes employed are altogether unnecessary. Only a certain amount of the medicine can find access into the system from the alimentary canal, and all the rest is either inert, or only a source of irritation. It is not necessary to interrupt the treatment already detailed. Calomel, or the mercurial pill, may be given in alternation, or conjointly, with the other remedies. The former preparation is best adapted to the early stages of the disease. From half a grain to two grains may be given every hour, two, or three hours, according to the urgency of the symptoms, and the known susceptibility of the patient. Let not those who are accustomed to enormous doses of this medicine, smile at those here mentioned. Let them first try even the smallest dose, in a sufficient number of cases, and determine, from the results of their experience, whether it may not be sufficient. When the stomach is very irritable, doses of only one-sixth of a grain, given every half hour or hour, and regularly persevered in, will often have a more decided constitutional effect than fifty times the quantity. The influence of these small doses, in calming bilious vomiting, is often most delightful. Should the bowels be very irritable, it will generally be advisable to combine the calomel with opium, and especially in the advanced stages. The medicine should be given regularly through the day; but should be omitted during six or eight hours at night, in order that the rest of the patient may be as little disturbed as possible. It is often proper to administer a somewhat larger than the ordinary dose at bedtime, with a grain or two of opium and the same quantity of ipecacuanha, if these be not contra-indicated, the former by the state of the brain, and the latter by irritation of stomach. The refreshing effects of sleep are thus obtained, and the patient frequently awakens in a fine perspiration in the morning.

"Calomel is very frequently administered in combination with tartar emetic and nitre, in the form of nitrous powders. This is no doubt often an efficient remedy; but all its effects may be obtained more agreeably, and less offensively to the stomach, by the mercurial, alternating with the effervescing draught, as before recommended. When the system obstinately resists the mercurial impression, or the case is very urgent, recourse should be had also to the external use of the remedy, in the form of the mercurial ointment, which may be applied by friction, or to blistered surfaces.

"The effects of mercury upon the gums should be watched for with care; and, as soon as any evidence of its action is afforded, the remedy should either be diminished or suspended, unless in cases of an almost desperate character."

Sulphate of quinine is an all important remedy, but should not be administered when there is high arterial excitement, especially when there is a tendency to cerebral inflammation. The author has seen much harm done by neglect of this caution. He is not an advocate for the large doses given by some.

"But there are circumstances, in bilious remittent fever, which render quinia of the utmost value. When a paroxysm of great virulence has occurred, from which the patient has been saved only by the most strenuous exertions, and there is every reason to fear that a similar one will prove fatal, recourse should be had to the sulphate of quinia, in the remission, however imperfect or short it may be. When the fever has hitherto shown little or no tendency to remit, and the grade ofviolence is such that fatal results appear imminent, should the slightest remission show itself, and the symptoms not be those of cerebal inflammation or strong determination, the quinia should be poured in without stint. The more nearly a case approaches to the above extremes, the stronger is the indication for the use of the antiperiodic medicine. I am entirely confident that I have seen lives saved by this treatment, which must have been inevitably lost under any other. The quantity of quinia given in the remission, must be sufficient to bring the system under the influence of the medicine, if possible, before the period for the next paroxysm. So far as my observation has gone, from eighteen to twenty-four grains are sufficient, in any case not falling under the title of malignant or pernicious; and often less will answer. The doses must be regulated by the length of the remission. If this be short, they must be very large, and if of a few hours' duration only, the whole quantity must be taken in two or three doses. If the remission be long, the medicine should be equally distributed through it, care being taken that the whole shall have been administered two or three hours before the expected paroxysm. The reader must not be led into the error of supposing, that the dangerous paroxysms above alluded to are characterized by excessive febrile excitement. On the contrary, there is often no remarkable degree of heat of skin, headache, or strength of pulse. The condition is rather marked by an extreme general distress, which the patient finds it impossible to describe, incessant jactitation, a feeling of burning heat while the extremities may be cool to the observer, incessant vomiting, great frequency of pulse, &c. When these subside into a comparative calm, then is the moment for the administration of the quinia.

"Nor should we be content with preventing a single paroxysm. The disease may be of the double tertian type, or may have a tendency to pass from the quotidian into the tertian. The remedy should therefore be continued until two daily paroxysms have been prevented; after which the patient may be considered safe.

"In the more regular paroxysmal forms of remittent fever, should quinia fail, or its use not be deemed advisable, the succession may often be effectually broken by an *emetic*, administered so as to be in full operation at the time of the expected paroxysm. For this purpose, tartar emetic would probably be more effectual than ipecacuanha, though I have seen the latter answer the purpose effectually more than once. A *pair of blisters* upon the legs or arms, applied so as to be in full action at the time above referred to, will often have the same effect. This remedy was very much employed, before the introduction of quinia into use. *Sinapisms* operate in the same way. A full dose of opium, also, given in anticipation of the paroxysm, will occasionally prevent it. When employed for this purpose, it should be combined with an equal weight of ipecacuanha."—(Vol. i, p. 278.)

When enteric or typhoid symptoms appear, the author advises the mercurial pill in doses of a grain every hour or two through the day until the gums are affected. This may be combined with quinine and other articles according to circumstances. If, notwithstanding the mercurial influence, no amendment takes place, the oil of turpentine will be found efficient, given in doses of ten or twenty drops every two hours. Nitrate of silver may be used under the same circumstances. On the whole the treatment recommended for this stage is similar to that of typhoid fever itself, and we shall refer to it under that head.

The modification of miasmatic fever, commonly termed malignant or congestive, is treated separately under the head of *pernicious* fever. This term is preferred to the former, because it has not been generally applied to other diseases, and is less likely to lead to erroneous views of the nature of the affection.

Pernicious fever is most frequently intermittent, or approximates to that form, and though often quotidian, its most common type is the tertian. The symptoms vary according as the organic or animal functions are most prominently affected. In the former case, evidences of disease will be presented in the organs of digestion, respiration, circulation, calorification and secretion; in the latter, in the brain. Differences also exist according to the organic function on which the morbific cause appears to fall. Thus, its force may be spent more particularly on the heart, the alimentary canal, or the surface of the body in its function of secretion or calorification.

It is important to be able to distinguish this from the ordinary forms of miasmatic fever, and to know whether there are any symptoms in the preliminary paroxysm that prognosticate danger. It should be borne in mind that it may occur in any instance of bilious fever, so that the subsequent yielding of the pernicious symptoms to the remedies employed, or in the regular course of the disease, shall not be regarded as commencing convalescence, but rather as a preparation for a more vigorous onset. The symptoms indicative of danger in a preliminary paroxysm are thus named by the author:

"An unusual paleness or lividness of the face; an absence of rigors or a sense of chilliness, while the extremities are really cold, and a want of uniform heat after reaction; a disposition to copious or frequent vomiting and purging, with a sense of unusual weight or oppression at the epigastrium; an extraordinary frequency, feebleness, or irregularity of the pulse; much anxiety, restlessness, or tossing about of the limbs, or a disposition to faintness; considerable delirium or drowsiness; a prolongation of the cold stage, and a less degree of febrile excitement than might have been anticipated; and the continuance in the apyrexia of some mental confusion, sleepiness, faintness, or unusual anxiety and uneasiness; any of the above symptoms should be a sufficient warning to the practitioner, not to delay for a moment, the measures requisite for interrupting the paroxysm."—(Vol. i, p. 288.)

Concerning the nature of this fever, Dr. Wood discourses at some length. The pernicious character in his opinion cannot be referred to inflammation; this may co-exist with other phenomena, but in most cases constitutes no portion of the danger. It is not the congestion; for this is present in other diseases without any such results. We must look beyond the congestion.

"It is in the peculiar state of the innervation, that we are to look for the source at once of the symptoms and the danger. This does not consist in a universal prostration of the nervous power. On the contrary, while defective in relation to certain functions, it may be unimpaired in others. Let us apply this view to an explanation of the symptoms. In the first place, in relation to the cases of collapse, in which the organic functions are especially concerned. This is prominently characterized by a want of action in the capillaries and extreme arteries. Some suppose that these vessels are spasmodically contracted. There is no evidence whatever of the existence of such spasm. They collapse simply because they contain no blood, just as they collapse in death. All parts of the organism receive a certain supply of nervous influence, which is essential to the due performance of their functions. The extreme vessels are probably not less under that influence than other parts. Evidence of this fact is abundantly supplied by phisiology. In the pernicious fever, the innervation of the extreme vessels fails, and they cannot, therefore, perform their part effectively in the circulation. The blood enters them with difficulty, in their enfeebled state, and is carried through them very slowly. Hence the paleness; and hence also the lividness of the surface, owing to the stagnation of the blood. From the same approach to nervous death in these vessels, they allow the watery portions of the blood to ooze through them, almost as through dead membrane. Hence the profuse sweats. The coldness obviously arises from the languid circulation, and deficient change of blood. This condition of the capillaries may co-exist with considerable power in the heart; for the want of innervation is not necessarily equal in the whole circulation. But sometimes, the deficiency is experienced first and specially by the heart. In such cases, syncope or a tendency to it is the prominent symptom.

"The function of respiration sometimes suffers from the same deficiency of innervation. The pulmonary capillaries cease to carry forward the blood with the usual rapidity, and the due aëration does not take place. Hence the feeling of oppression of chest and want of breath, the deep sighing, &c. Some writers ascribe these phenomena to congestion of the lungs. They are the consequence of a want of proper decarbonization or oxidation of the blood, not of mere congestion. In the cases of Maillot, the lungs were found remarkably free from disease after death.

But how account for the oppression of stomach, and the copious vomiting and purging? Upon the same principles exactly. There is no feeling of the stomach, unless it may be excessive spasm, which is more insupportable than that arising from deficient innervation. This can be readily understood by those who have experienced the distressing sensations of a half paralyzed limb. The bloody serum, or pure blood, discharged from the stomach and bowels, escapes through the coats of the vessels, exactly as blood percolates through the tissues after death; with this exception, that, as the vis a tergo still continues in some degree, it adds a vital expelling force to that of mere physical transudation. The alimentary canal may be said to sweat, like the external surface. An appeal may be made to the discoloration, softening, &c., of the mucous membrane, as evidences of inflammation or congestion. I am not disposed to deny the existence of inflammation, in some degree in this membrane. But it is quite insufficient to account for the symptoms. We do not witness the same phenomena in other cases of much more extensive inflammation. Besides, much of the softening of the tissue may be ascribed to depressed vitality, and much of the discoloration to the stagnation of blood, like the livid or purple color of the skin.

"But the thirst and sense of internal heat; are they not signs of congestion or inflammation? By no means. Thirst is a common accompaniment of all conditions of disease in which the capillaries are emptied of their blood, and such is undoubtedly the source of it here. The heat can be referred only to morbid nervous influence. That there is no real increase of temperature within, is inferred from the state of the tongue. Besides, the sensation is not confined to the internal parts. I have known patients to complain, under these circumstances, of distressing heat of surface, when their skin was positively much below the healthy temperature. This is merely another example of morbid innervation. It appears, then, that this defect or derangement of innervation lies at the basis of all the morbid phenomena of the organic functions. The congestion necessarily follows the prostration of the active circulating forces. The pulmonary capillaries, the heart, and the systemic capillaries are all enfeebled; the blood, therefore, collects in the veins and in the great internal organs, especially in those connected with the portal circulation. Hence the congestion of the liver and spleen. When the circulatory movements return with their wonted activity, under the restored innervation, the congestion is speedily dissipated.

"In the cerebral cases, the deranged innervation is experienced primarily and chiefly in the lobes of the brain. The organic functions are at first comparatively unaffected, the heart often continuing to act with energy, and the surface to retain its warmth, when the patient is quite insensible. That the affection is chiefly nervous, is to be inferred from its periodicity. There may often be congestion; there may sometimes be inflammation of the brain. But the presence of an excess of blood is probably due less to increased determination, than to stagnation in the capillaries. The color of the cortical portion is described by M. Maillot as approaching to black. The brain differs from other organs in the circumstance of occupying a closed cavity, into which atmospheric pressure alone would force the blood, so that the feebleness of the capillaries cannot here be followed by paleness as upon the surface. The vessels must always be full, or the vacuity must be compensated by effusion. Redness, therefore, and serum in the ventricles are not necessarily signs of over-excitement. But I am not disposed to maintain, that the cerebral affection in these cases is certainly the result of diminished sensorial action. It may be so; or it may proceed from some other derangement of that action. All that I would maintain is, that the affection is essentially nervous; and that upon this the danger depends. The patient dies, not because he has too much blood in his brain, but because the brain becomes incapable, from the direct influence of the morbid cause, of performing the functions essential to life. This question is not merely speculative. It is on the contrary highly practicable; and its decision, one way or the other, may greatly influence the treatment. If the disease be considered in the light of apoplexy or cerebritis, copious bleeding would suggest itself as the appropriate remedy; if it be nervous, we must look for safety to some means capable of strongly impressing the nervous system."—(Vol. i, 290, '91, '92.)

The indication, if the patient is seen in the paroxysm, is to bring about reaction as soon as possible. This is to be effected not by bleeding and large doses of calomel, as formerly recommended and practiced, but by artificial heat, and stimulants applied externally, and largely given internally. Where the brain is seriously involved, with a tolerably strong pulse, a vein may be opened in the arm, and cups or leeches applied to the temples; but in no other case is bleeding proper. Where there is excessive vomiting, or exhausting diarrhœa, or hemorrhage, opium and the astringents must be used along with the stimulants. Quinine may also be given during a paroxysm, for its excitant influence on the nervous centres, and also in reference to the next paroxysm. The application of cold water by affusion is favorably spoken of by Dr. Wood. But as soon as an intermission or remission has been obtained there is but one course of treatment. Sulphate of quinine must be given without delay at any and all hazards. If the apyrexia is short, the doses must be large; if the apyrexia is longer, the doses may be smaller; in both cases they should be given so that the whole quantity may be swallowed two or three hours before the time for the recurrence of the paroxysm.

Typhoid fever we find discussed at some length. The author does not regard it as contagious, or at least but feebly so. We cannot follow him in his description of the symptoms, but we subjoin the diagnosis.

"The most characteristic symptoms of this disease are the frequently slow and insidious mode of attack, the diarrhea at the beginning or soon afterwards, the dull or heavy expression of countenance, the dusky hue of the face, the tendency to epistaxis, the cough and bronchial rales, and, after the seventh or ninth day, the dryness of the tongue and general diminution of the secretions, the rose-colored eruption, the sudamina, the tympanitic abdomen, the deafness, the stupor or delirium, and the various signs indicative of the typhus state. The enlargement of the spleen, either sensible to the touchor discoverable by percussion, may have some weight in the diagnosis. The duration of the disease, exceeding generally that of other fevers, is also an important diagnostic character. It must not, however, be understood, that all these symptoms are necessarily present in every case. The diagnosis may be very certain, though many of them should be absent.

"One of the diseases with which enteric fever is most frequently confounded is the remittent or bilious fever. The latter, however, may usually be distinguished by its more regular and decided remissions, by the bilious vomiting and yellowness of skin which frequently attend it, by its shorter duration and its tendency to end in intermittent, and by the absence or rarity of the characteristic symptoms of enteric fever, such as diarrhœa, epistaxis, dingy complexion, hebetude of expression, stupor, tympanites, and rose-colored spots. Typhus symptoms are much less common in bilious than in enteric fever. Dissection confirms the diagnosis. While in bilious fever the stomach is more frequently inflamed, and the liver discolored than in the enteric; the spleen is less diseased, and there is a total absence of the affection of the glands of Peyer, and of the mesenteric glands, characteristic of the latter complaint. Sometimes, however, the diagnosis is very difficult, especially when the bilious fever assumes the protracted typhoid form. I believe that, occasionally, the two complaints are commingled, in consequence of the co-operation of their causes. Thus, cases having all the essential characters of enteric fever occasionally end in intermittent; and bilious fevers, or affections which cannot be distinguished from them, sometimes show the signs of enteric fever during their progress."—(Vol. i, pp. 329, '30.)

From typhus fever it cannot always be clearly distinguished. The author suggests that now and then the two diseases exist conjointly. The most important points of difference are thus stated.

"Typhus fever less frequently commences insensibly than the enteric, and is, upon the average, of considerable shorter duration. Instead of the diarrhœa, or extraordinary susceptibility to the action of purgatives, which attends the latter disease, it is usually characterized by constipation; and when fecal discharges are obtained, they are usually darker and more offensive. But hemorrhage from the bowels, which is not unfrequent in the advanced stages of enteric fever, seldom occurs in typhus. In the latter complaint, epistaxis at the commencement is less frequent; there is more stupor and a darker color of the face, more turbidness of the conjunctiva, and much greater debility. The eruption in typhus also differs from that of enteric fever. It generally commences earlier; is not elevated as the other; is less regularly round or oval, is of a darker, more livid hue; does not so readily disappear under pressure, and is often unaffected by it; is much more abundant; and, instead of being confined chiefly to the abdomen and chest, is found equally upon the extremities, and is often diffused over almost the whole body. In typhus fever the abdomen is often flat, and perfectly free from tympanites, which is never the case in the enteric. The signs of consolidation of the posterior part of the lungs are much more freqvently present in the former, and the dry sabilant rales of bronchial inflammation in the latter.

"The anatomical characters of the two diseases are very different. The peculiar disease of the glands of Peyer, and of the mesenteric glands, so constantly present in enteric fever, is never found in typhus, or so seldom as to lead to the suspicion of some intermixture of diseases when it does occur. The spleen, too, is much less frequently enlarged and softened in the latter.

"There are other points of difference in the persons attacked, and the circumstances under which the diseases originate.

Thus, enteric fever almost never attacks the old, who are very frequent victims of typhus. The former disease is endemic in various countries, arises here and there (sporadically) without any obvious cause, and, if ever contagious, is very feebly and very rarely so; while typhus seldom occurs in isolated cases, is often long absent from countries where it occasionally prevails, is always contagious, and often epidemic."—(Vol. i, p. 346.)

According to the author typhoid fever is not a very fatal disease. As the result of his own observation few serious diseases exhibit more happily the controlling influence of treatment. Though the complaint cannot be suddenly interrupted, yet it may be materially shortened, and often conducted to a favorable issue, when without treatment it would end in death. "No case," says Dr. Wood, and we beg the reader to mark it, "no case should be looked upon as absolutely desperate. There is no condition so low, no symptom so fatal that death should be considered inevitable. It is only in articulo mortis that the case should be given up." But the prognosis must always be cautious, as no case, however light it may be, can be regarded as absolutely free from danger. Intestinal perforation, the most desperate of all the symptoms, is liable to occur in the mildest cases.

The treatment recommended can be stated in a few words. In the beginning, and throughout the disease, there should be thorough evacuation of the bowels by the milder purgatives, or, if the stomach is irritable, by enemata. Often this is not necessary, as the evacuations are spontaneous and free. Bleeding in many cases is wholly uncalled for. It will not arrest the disease, and as this is apt to be protracted, the strength of the patient must be husbanded. As in remittent fever, bleeding is useful only to prevent or control local and disorganizing inflammation. When this is not present, and the pulse is not full and strong, it is better to omit it. When employed it should be in the first four or five days. Local bleeding by cups and leeches may be employed at any stage, unless the debility is very great, to obviate local inflammation or sanguineous determination. Refrigerant diaphoretics, as in the remittent form, are useful from the commencement. The citrate of potassa, in the form of neutral mixture or effervescing draught, is a favorite with the author. It may be combined with a little antimony, when the pulse is pretty firm and the stomach is not irritable; or, to fulfil other indications, with laudanum or some other preparation of opium. Cold ablutions, or sponging of the legs, arms, and temples, will be useful. Local affections will require attention: obstinate headache must be treated by leeches, and cold water or ice in bladders; tenderness in the abdomen by leeches or cups, and large mush poultices steadily applied day after day—sometimes a blister; diarrhœa, if exhausting, by small doses of opium and ipecacuanha, with acetate of lead, rhatany, tannic acid, &c.

This treatment, in the more favorable cases, will frequently prove sufficient. But very often about the seventh or ninth day, while there is no diminution in the violence of the disease, the energy of the vital actions has abated. There is a general deficiency of secretion, the tongue is dry, the skin dry, and the urine scanty. The pulse is more frequent, but less strong and full. There may be delirium or increase of stupor, and tympanites. Now is the time for disorganizing inflammation; and to prevent or cure this, and restore the suspended secretions, there is no remedy equal to mercury in minute doses, continued until the gums are slightly touched. Dr. Wood prefers the blue mass, as the impression must be made in the mildest way. Should the symptoms not yield, especially if the tongue remains dry and the abdominal distention undiminished, the oil of turpentine is strongly recommended by him.

"I cannot too strongly impress upon the profession my convictions of the importance of this medicine. It may be employed in all cases, in the advanced stages of this disease, when the tongue is dry, and the pulse not strong. But there is a particular condition, and that a not uncommon and sometimes a very dangerous condition, in which I have often employed it, and hitherto have in no one instance known it to fail. In the description of the symptoms, it was stated, that, in the latter period of the disease, the tongue, instead of cleaning gradually from the edges and tip, often parts with its fur quickly and in large flakes, generally first from the middle or back part of the surface, which is left smooth and glossy, as if deprived of its papillæ. It was also stated, that if, after this process, the tongue remain moist, a slow convalescence may be pretty confidently calculated on. But it not unfrequently happens, that, during the progress of the cleaning process, or after its completion, the surface of the tongue becomes quite dry, and the process, if not finished, is suspended. At the same time, there is generally an increase of the tympanites, and an aggravation, or certainly no abatement, of the other symptoms. Two cases of this kind I had seen terminate fatally, one in my own practice, and a second in that of a medical friend. One of them was examined after death; and ulcers were found in the ileum near the illeo-cœcal valve. This case happened so early as the year 1823. Ascribing the aggravation of symptoms which attended the drying of the tongue, after cleaning, to the occurrence of ulceration in the ileum, I inferred that the oil of turpentine, which had been recommended in ulcerations of the intestines, might prove useful here, and determined to employ it in similar cases. I did so, and, as before stated, have never yet found it to fail, under the circumstances mentioned, though I have had frequent occasion to resort to it, both in private and public practice. It acts in some measure as a stimulant, but chiefly, I believe, as an alterative to the ulcerated surfaces in the intestinal mucous membrane. It should be given in doses of from five to twenty drops every hour or two, and is best administered in emulsion with gum arabic, loaf sugar, and water; a little laudanum being occasionally added, if it disturb either the stomach or the bowels. In the course of twenty-four, or at most forty-eight hours, some amelioration of the symptoms may be observed. The tongue becomes gradually moister, and covers itself with a whitish fur; the tympanitic distension ceases to augment, and after a time diminishes; the pulse becomes less frequent, and the skin less dry and harsh; and the patient enters slowly but regularly into convalescence, often without any other remedy. As the case improves, the quantity of the oil should be diminished; but care should be taken not to omit it too hastily."—(Vol. i, pp. 333, '34.)

We have filled our allotted space without having noticed half of the subjects marked for that purpose. We assure the reader that he will find in this treatise a body of sound precepts, clearly, correctly, and even elegantly expressed. C.

